# Demographic Analysis of Cancer Research Priorities and Treatment Correlations

**DOI:** 10.3390/curroncol31040139

**Published:** 2024-03-29

**Authors:** Denis Horgan, Marc Van den Bulcke, Umberto Malapelle, Nicola Normanno, Ettore D. Capoluongo, Arsela Prelaj, Carmelo Rizzari, Aliki Stathopoulou, Jaya Singh, Marta Kozaric, France Dube, Manuel Ottaviano, Stefania Boccia, Gabriella Pravettoni, Ivana Cattaneo, Núria Malats, Reinhard Buettner, Karim Lekadir, Francesco de Lorenzo, Catherine Alix-Panabieres, Sara Badreh, Eric Solary, Ruggero De Maria, Paul Hofman

**Affiliations:** 1 European Alliance for Personalised Medicine, 1040 Brussels, Belgium; jayasinghtec29@gmail.com (J.S.); marta.kozaric@euapm.eu (M.K.); 2 Department of Molecular and Cellular Engineering, Jacob Institute of Biotechnology and Bioengineering, Faculty of Engineering and Technology, Sam Higginbottom University of Agriculture, Technology and Sciences, Prayagraj 211007, India; 3 Belgian Cancer Centre, Sciensano, 1050 Brussels, Belgium; marc.vandenbulcke@sciensano.be; 4 Department of Public Health, University Federico II of Naples, 80138 Naples, Italy; umberto.malapelle@unina.it; 5 Istituto Nazionale Tumori “Fondazione G. Pascale”—IRCCS, 80131 Naples, Italy; n.normanno@istitutotumori.na.it; 6 Department of Molecular Medicine and Medical Biotechnologies, University of Naples Federico II, 80138 Naples, Italy; edotto70@gmail.com; 7 Department of Clinical Pathology, Azienda Ospedaliera San Giovanni Addolorata, Via Amba Aradam 8, 00184 Rome, Italy; 8 Department of Medical Oncology, Fondazione IRCCS Istituto Nazionale Tumori, 20133 Milan, Italy; arsela.prelaj@istitutotumori.mi.it; 9 Unità di Ematologia Pediatrica, Fondazione MBBM, Università di Milano-Bicocca, 20126 Monza, Italy; carmelo.rizzari@gmail.com; 10 European Cancer Patient Coalition, 1000 Brussels, Belgium; aliki.stathopoulou@ecpc.org (A.S.); francesco.delorenzo@ecpc.org (F.d.L.); 11 Astra Zeneca, Concord Pike, Wilmington, DE 19803, USA; france.dube@astrazeneca.com; 12 Departamento de Tecnología Fotónica y Bioingeniería, Universidad Politècnica de Madrid, 28040 Madrid, Spain; manuel.ottaviano@upm.es; 13 Section of Hygiene, Department of Life Sciences and Public Health, Università Cattolica del Sacro Cuore, 20123 Rome, Italy; stefania.boccia@unicatt.it; 14 Departments of Woman and Child Health and Public Health, Fondazione Policlinico Universitario A. Gemelli IRCCS, 00168 Rome, Italy; 15 Department of Oncology and Hemato-Oncology, University of Milan, 20122 Milan, Italy; gabriella.pravettoni@ieo.it; 16 Applied Research Division for Cognitive and Psychological Science, European Institute of Oncology (IEO) IRCCS, 20139 Milan, Italy; 17 Novartis Farma SpA, 21042 Origgio, Italy; ivana.cattaneo@novartis.com; 18 Genetic and Molecular Epidemiology Group, Spanish National Cancer Research Centre (CNIO), 28029 Madrid, Spain; nmalats@cnio.es; 19 Lung Cancer Group Cologne, Institute of Pathology, Center for Integrated Oncology Cologne/Bonn, University Hospital Cologne, 50937 Cologne, Germany; reinhard.buettner@uk-koeln.de; 20 Artificial Intelligence in Medicine Lab (BCN-AIM), Universitat de Barcelona, 08007 Barcelona, Spain; karim.lekadir@ub.edu; 21 Laboratory of Rare Human Circulating Cells, University Medical Center of Montpellier, 34093 Montpellier, France; c-panabieres@chu-montpellier.fr; 22 Cancer Childhood International, 1200 Vienna, Austria; s.badreh@ccieurope.eu; 23 INSERM U1287, Gustave Roussy Cancer Campus, 94805 Paris, France; eric.solary@gustaveroussy.fr; 24 Faculty of Medicine, Université Paris-Sud, 91405 Le Kremlin-Bicêtre, Île-de-France, France; 25 Department of Hematology, Gustave Roussy Cancer Campus, 94805 Paris, France; 26 Institute of General Pathology, Catholic University of the Sacred Heart, 20123 Rome, Italy; presidenza@alleanzacontroilcancro.it; 27 IHU RespirERA, FHU OncoAge, Laboratory of Clinical and Experimental Pathology, Pasteur Hospital, Université Côte d’Azur, 06000 Nice, France; hofman.p@chu-nice.fr

**Keywords:** cancer, research, demographics, policy, personalized medicine, priorities, patients, oncology

## Abstract

Understanding the diversity in cancer research priorities and the correlations among different treatment modalities is essential to address the evolving landscape of oncology. This study, conducted in collaboration with the European Cancer Patient Coalition (ECPC) and Childhood Cancer International-Europe (CCI-E) as part of the “UNCAN.eu” initiative, analyzed data from a comprehensive survey to explore the complex interplay of demographics, time since cancer diagnosis, and types of treatments received. Demographic analysis revealed intriguing trends, highlighting the importance of tailoring cancer research efforts to specific age groups and genders. Individuals aged 45–69 exhibited highly aligned research priorities, emphasizing the need to address the unique concerns of middle-aged and older populations. In contrast, patients over 70 years demonstrated a divergence in research priorities, underscoring the importance of recognising the distinct needs of older individuals in cancer research. The analysis of correlations among different types of cancer treatments underscored the multidisciplinary approach to cancer care, with surgery, radiotherapy, chemotherapy, precision therapy, and biological therapies playing integral roles. These findings support the need for personalized and combined treatment strategies to achieve optimal outcomes. In conclusion, this study provides valuable insights into the complexity of cancer research priorities and treatment correlations in a European context. It emphasizes the importance of a multifaceted, patient-centred approach to cancer research and treatment, highlighting the need for ongoing support, adaptation, and collaboration to address the ever-changing landscape of oncology.

## 1. Introduction

**Figure 1:**
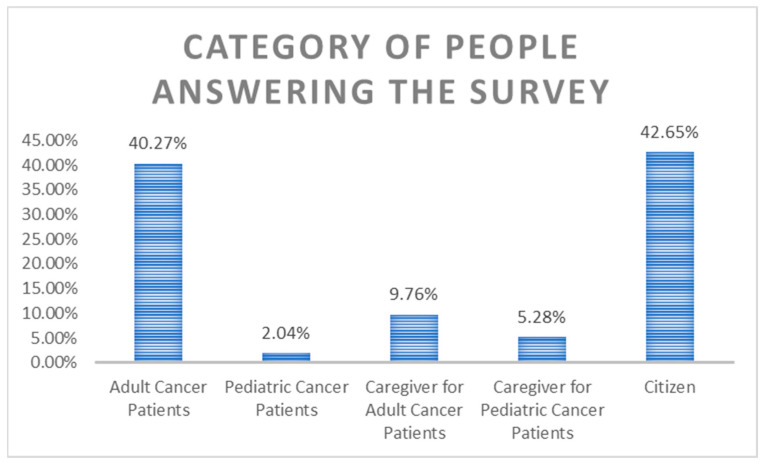
Category of survey participants.

Cancer stands as a prominent contributor to mortality, with its occurrence consistently on the rise globally, primarily driven by the ageing of the population (Figure 1). Diverse factors contribute to significant disparities in outcomes among nations, including variations in cancer types, national cancer screening policies, initial stages at diagnosis, availability of high-quality treatments such as radiotherapy and systemic therapy, and cultural obstacles. Survival depends on access to appropriate treatment, which in turn is linked to the economic capability and healthcare policy of individual countries [1,2]. Cancer is responsible for one in seven deaths worldwide, surpassing the combined fatalities from HIV/AIDS, tuberculosis, and malaria [3]. The World Health Organization projects mortality rising to 15 million new cases by 2020 [4]. In Brazil alone, an estimated 420,000 new cases emerged in 2016–2017, excluding non-melanoma skin cancer [5]. The spectre of death looms over every stage of the cancer experience, from diagnosis through treatment and beyond, as suggested by the Terror Management Health Model [6] and this perception of cancer can impact early diagnosis, screening program adherence and treatment decisions. The elderly (60 years of age or older), with their elevated risk factors and compromised immune systems, face a particularly daunting challenge, with 70% of cancer cases in the United States projected to occur in this age group by 2030 [7]. Understanding how elderly patients perceive cancer is pivotal for predicting their health-related behaviours and informing tailored strategies for health education and cancer prevention [8].

Different demographic groups have varied perceptions of the importance of cancer research and priorities for research funding, in Europe and elsewhere, informed by personal experiences, cultural influences, and societal priorities [9]. Rural areas are often areas of health workforce shortage, and they have reduced health infrastructure and higher costs of health care delivery. Across many health conditions, there is a health disadvantage in rural areas. The rates of smoking are higher in rural areas and people in rural and remote areas might experience more problems in accessing and following advice that is relevant to prevention [10,11]. Success largely depends on assessing whether needs differ among types of healthcare practice, and identifying the specific areas in preventive services where clinicians require the most improvement and assistance [12]. In the past decade, close to 100 new cancer medications have gained approval in Europe, but their availability is limited, with Central and Eastern European (CEE) countries notably trailing Northern and Western Europe. This reflects variations in reimbursement rates, with 64% of indications being reimbursed in Czechia, 40% in Hungary, 51% in Poland, and 19% in Slovakia. The increasing expenditure on cancer drugs in Europe has made it progressively challenging to provide access where resources are low, necessitating a prioritized approach to ensure access [13,14,15]. 

Despite well-documented variations in treatment approaches, there is a paucity of research investigating how individuals of different age groups—namely, older, middle-aged, and younger adults diagnosed with cancer—make choices regarding their treatment options [16]. There are also few studies exploring the distinctions in the decision-making process across these age groups. It is important to recognize that patients’ decisions about cancer treatment are not solely the outcome of a rational evaluation, but are influenced by circumstances surrounding the diagnosis, interpersonal interactions, and individual determinants, in addition to the medical prognosis. In geriatric oncology, striking a balance between the potential risks and benefits of treatment is all the harder because of the limited data on survival rates and the patients’ quality of life. Older adults diagnosed often suffer multiple comorbidities, cognitive impairments, and polypharmacy medications, all of which can further complicate decisions over treatment options [17].

The COVID-19 pandemic had serious consequences for cancer patients. Stay-at-home recommendations led to delayed diagnoses, more advanced cases, changes in treatment guidelines, and poorer outcomes [18,19]. COVID-19 safety guidelines were established in cancer centers, but the pandemic’s long-term impact on cancer progression, morbidity, and mortality remained underexplored. This was demonstrated by a study from Brazil that recorded significant drops in hospital admission rates for clinical and surgical cancer treatments, and compared the numbers for 2020 with those in 2018, 2019, and 2021 for newly diagnosed single and multiple cases, cancer stage at diagnosis, and times to treatment initiation [9].

Pediatric cancers impose a somber burden on the lives of numerous children and their families, standing as a prominent cause of mortality among children globally. Despite notable progress in medical research and treatment approaches, addressing pediatric cancers remains an arduous and pressing imperative [20,21].

Patient and public involvement (PPI) in healthcare and research is on the rise in Europe, reflecting a growing interest and support for this approach [22]. This is characterized by collaborations between academics and patients, and by the emergence of patient-inclusive journals, such as the Patient Experience Journal, which holds the Patients Included™ status, signifying active participation of patients in editorial boards, authorship, peer review, and ensuring open access. The inclusion of patients in research is rooted in several compelling arguments. One key argument is that patients possess a unique form of knowledge derived from their lived experiences with illness, vulnerability, or disability [23]. This experiential perspective is often referred to as the ‘emic’ or ‘insider’ view, complementing the ‘etic’ or ‘outsider’ perspective of professionals. It is seen as an invaluable enrichment of the societal impact and relevance of research. Another argument is based on the principles of epistemic justice, where patients, as end-users, should have a voice in research that directly impacts their lives, in line with the notion of the fundamental human right to be acknowledged as bearers of knowledge [24].

But implementing PPI is less straightforward, as testified by studies revealing inconsistent adoption, with integration low in European healthcare systems and research. Patients enjoy only limited influence over the research processes in which they are involved, often merely informed about the study, consulted occasionally through interviews or focus groups, and very rarely included in steering groups—to the disadvantage particularly of patients living in vulnerable situations, a group often termed the ‘seldom heard’ [25]. Academics still dominate as initiators, shaping research agendas and retaining control. Involving patients in research runs up against entrenched power hierarchies in healthcare practices, in which professional caregivers and researchers often enjoy a privileged epistemic status based on their expertise. Inadvertently, patients’ narratives and interpretations are neglected. The existence of negative stereotypes in healthcare, particularly in psychiatry and chronic illness, can lead to the underestimation of the testimonies of patients who may not fit the traditional medical model, or who face judgments regarding their intelligence and credibility based on their language skills and discourse [26,27].

The recent development of frameworks for PPI and patient-led research agenda-setting aims to recognize patients’ priorities as of equal importance to those of professionals and care providers. They often focus on specific diagnoses or patient groups and challenge the traditional dynamics of healthcare research. However, a gap remains in understanding how to co-create such frameworks and which topics should take precedence [28]. The goal of the study was to assess how age-specific perspectives influence cancer research priorities, prevention strategies, early detection initiatives, treatment approaches, and healthcare resource allocation, and what are the regional and country-specific variations within Europe.

## 2. Materials and Methods

This research aimed to investigate the diversity in cancer research priorities across European member states and among different stakeholders based on demographics (age and gender), the time since first diagnosed with cancer, and the treatments received. The study was conducted in collaboration with the European Cancer Patient Coalition (ECPC) and Childhood Cancer International-Europe (CCI-E) as part of the broader “UNCAN.eu” initiative [29,30].

### 2.1. Data Collection

Data was collected through a comprehensive survey conducted by the ECPC and CCI-E, with analysis conducted by the European Alliance for Personalised Medicine (EAPM). The survey was designed to gather insights into cancer research priorities from diverse groups, including adult cancer patients, cancer survivors, caregivers, pediatric cancer patients, and individuals not directly affected by cancer. The survey encompassed a wide range of demographic variables, time since diagnosis, and treatment experiences (please see the Supplementary Materials).

### 2.2. Survey Design

The survey was designed to gain an idea of European citizen’s interest and priorities in cancer research. It was developed (on the SurveyMonkey platform), conducted and validated by the European Cancer Patient Coalition (ECPC) and the Childhood Cancer International Europe (CCI-E). The questionnaire covered six research topics previously identified by the Expert Working Groups in WP2, and also had questions on Data sharing:

(1) Factors Influencing Cancer Development and Risk
(2) Cancer Prevention and Early Detection
(3) Cancer Biology and Therapeutic Approaches
(4) Aging and its Intersections with Cancer
(5) Cancer Complications and Survivorship
(6) Data Generation and Utilization in Cancer Research

These 35 measures were identified under the six major pillars (Table 1).

Survey participants were asked to indicate cancer research areas that they find important for the EU Commission to prioritize for the design of new policies, budgets, and resources. They could indicate the areas and following topics within each research area in a scale from 1 to 5, where 1 is “not important at all” and 5 is an “absolutely important”.

To allow participants to develop an informed opinion, the survey included background information about each topic and associated research areas. To increase the level of participation across Europe and get the most representative result, the survey was translated from English into the following 12 languages by native-language partners and colleagues: Bulgarian, Dutch, French, German, Greek, Hungarian, Italian, Polish, Portuguese, Romanian, Slovak, Spanish.

The questionnaire was anonymous with no personal information and identification details of the participants being recorded. Participants’ information was adequately protected against access by third parties and processed by the provisions of the General Data Protection Regulation (GDPR).

The survey was distributed through professional, social, and scientific networks (websites and social media) with the support of the whole consortium. The data collection took place from 20 November 2022 to 20 February 2023. 

In total, 1768 responders initiated the survey, while a total of 1350 participants with complete responses were obtained.

#### Survey Participants

The survey responders were EU citizens not directly affected by cancer (42.6%), adult cancer patients (40.3%), caregivers for adult cancer patient (9.8%), caregivers/parents for pediatric cancer patient (5.3%), and pediatric cancer patient (2%) (Figure 1). 

**Figure 2:**
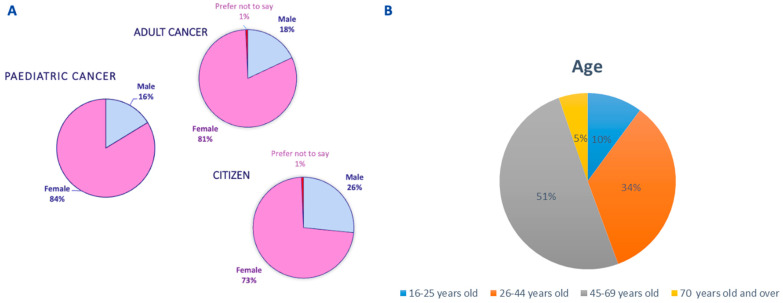
Demographics of study participants: (**A**) gender and (**B**) age.

For further analysis the survey participants were pooled into three groups: adult cancer patients (adult cancer patients and their caregivers); pediatric cancer patients (pediatric cancer patients and their caregivers/parents), and EU citizens (participants not directly affected by cancer). The majority of survey responders were women (average within groups 79%), aged between 26–44 in pediatric cancer patient and EU citizen groups, and between 45–69 years old in adult cancer patient group (Figure 2).

Participants from various European countries took part in the study, with the following numbers from each nation: Belgium contributed 59 participants, Bulgaria contributed 56, France had 136, Germany had 95, Greece had 35, Hungary had 141, Italy had 338, Luxembourg had 38, the Netherlands had 61, Portugal had 102, Romania had 33, Slovakia had 63, and Spain had 119. The inclusion of a diverse range of countries and participant numbers ensured a broad and comprehensive representation in the study, allowing for a more nuanced understanding of demographic factors and their impact on cancer research priorities across Europe.

### 2.3. Demographic Data

Demographic information collected included age and gender. Age groups were categorized as 16–25, 26–44, 45–69, and over 70 years.

### 2.4. Time since First Diagnosis

Data was collected on the time elapsed since the first cancer diagnosis, categorized into four groups: 1 year, 1–3 years, 4–10 years, and more than 10 years.

### 2.5. Treatment Data

Participants were asked about the types of cancer treatments they received post-diagnosis, including surgery, radiotherapy, precision therapy, chemotherapy, biological therapies, hormone therapy, and other therapies.

### 2.6. Statistical Analysis

To examine the associations among different factors, such as demographics, time since diagnosis, and treatment received, we initially standardized all the data on a 100-point scale for consistency. Subsequently, we performed statistical analyses on the normalized data. This analysis aimed to quantify the extent and direction of associations between different variables.

#### 2.6.1. Correlation Analysis

Correlation is a fundamental statistical measure used to evaluate the extent to which two variables change together. In this study, correlation coefficients were calculated to determine the strength and nature of relationships between various data sets. The Pearson correlation coefficient, commonly known as Pearson’s r, was employed. This coefficient quantifies the linear relationship between two continuous variables and ranges from −1 to 1. The interpretation of the Pearson correlation coefficient is as follows:

A value of 1 indicates a perfect positive correlation, signifying that as one variable increases, the other also increases linearly.

A value of −1 denotes a perfect negative correlation, indicating that as one variable increases, the other decreases linearly.

A value of 0 suggests no linear correlation between the variables. It signifies that changes in one variable are not associated with changes in the other in a linear fashion.

#### 2.6.2. Ranking

Ranking of data points was employed as a part of the analysis. This process involves arranging data points from highest to lowest (or vice versa) based on specific criteria, such as the “Measure” values for each cancer type within a particular measure or pillar. The highest value is assigned a rank of 1, the second-highest receives a rank of 2, and so on. If multiple data points share the same value, they are assigned the same rank, with the subsequent rank(s) being skipped.

#### 2.6.3. Percentiles

Percentiles were calculated to provide a more detailed understanding of the distribution of data values within a set. The percentile of a data value indicates the percentage of data values in the set that fall below that particular value. To calculate the percentile of a given data value, the following formula was employed:

n = (P/100) × N

where:

N represents the total number of values in the data set.

P represents the desired percentile.

n represents the ordinal rank of a specific value within the data set when the values are sorted from smallest to largest.

These statistical measures allowed for a comprehensive analysis of the data, facilitating the understanding of patterns and associations between various factors and research priorities. It is essential to note that correlation does not imply causation, meaning that identified relationships do not necessarily indicate a cause-and-effect connection between variables.

## 3. Results

### 3.1. Demographic Analysis

#### 3.1.1. Correlation among Age Groups

- 16–25 vs. 26–44 Years:

A moderate positive correlation of 0.738 suggests that individuals aged 16–25 years and those aged 26–44 years share some commonalities in their cancer research priorities. This implies that certain themes or areas of interest in cancer research persist as individuals transition from the younger age group to the early adulthood stage.

- 26–44 vs. 45–69 Years:

A strong positive correlation of 0.935 indicates a highly aligned research focus between individuals aged 26–44 years and those in the 45–69 age group. This suggests a continuity and strengthening of research priorities as individuals move from mid-adulthood to the later stages of adulthood, indicating potentially shared concerns or challenges in cancer research across these age ranges.

- 45–69 vs. Over 70 Years:

A very strong positive correlation of 0.979 reflects a highly aligned research focus within the 45–69 age group. However, the correlation decreases to approximately 0.416 among individuals over 70 years and 16–25 years, signaling a divergence in research priorities compared to the younger age groups. This divergence might be attributed to age-related differences in health concerns, treatment options, or research interests. 

#### 3.1.2. Correlation among Gender

- Males vs. Females:

A strong positive correlation of 0.769 indicates a high level of alignment in research focus between males and females. This suggests that, despite potential biological or gender-specific factors, the overall priorities in cancer research are notably consistent across both genders. This shared focus underscores common concerns or universal aspects of cancer research. (Table 2).

#### 3.1.3. Rank and Percentile

The survey data on cancer research priorities offers a comprehensive view of the preferences and priorities among respondents across different demographic groups. One significant observation is the clear variation in research preferences across age groups. Measures 11, 9, 10, 16, and 17 consistently receive the highest rankings across all age categories. These measures predominantly emphasize areas such as gut bacteria, genetics, and early detection. This implies that regardless of age, respondents consider research related to these aspects as a top priority in the fight against cancer. Such a consensus across age groups is vital for guiding research strategies, indicating that these areas require continued and possibly expanded attention.

Moreover, the data highlights gender-based differences in research priorities. Measure ID 10, focused on “Screening and Early Detection,” emerges as a common priority for both males and females. While many other measures demonstrate gender-specific variations, this shared priority underscores the significance of early detection and screening initiatives in cancer research. Understanding these gender-based differences can help in developing targeted awareness and screening programs, ensuring that the needs and preferences of both males and females are met effectively.

Beyond age and gender, the data provides insights into how specific cancer research measures rank across different demographics. For instance, measures focusing on novel therapies and treatment options receive varying degrees of attention depending on the demographic group. Understanding these variations can help research institutions, funding bodies, and policymakers allocate resources more effectively. This means that research efforts should not only concentrate on popular areas but also explore less prioritized ones to ensure a comprehensive approach to cancer research (Table 3).

In conclusion, the survey data on cancer research priorities paints a nuanced picture of what various demographic groups believe should be the focus of cancer research. The consensus among different age groups on certain research areas underscores their critical importance. At the same time, recognizing gender-based differences can lead to more targeted research and healthcare interventions. These insights provide a roadmap for the allocation of resources and funding, ensuring that cancer research is both comprehensive and responsive to the diverse needs of the population. Researchers, policymakers, and healthcare professionals should take these priorities into account when developing strategies to combat this complex and challenging disease.

### 3.2. Time First Diagnosed

The correlation table presents insightful patterns in cancer research priorities across various time intervals since patients were diagnosed with cancer. In the first year after diagnosis, the correlation of 1.000 indicates a strong, positive relationship in research priorities during this critical initial period. As patients move into the 1–3-year timeframe post-diagnosis, the correlation remains notably high at 0.9318, signifying a continued alignment in research foci (Table 4). This suggests a consistent emphasis on specific research areas during the early years after diagnosis.

As the post-diagnosis period extends to 4–10 years, the correlations of 0.8526 (with the first year) and 0.8952 (with 1–3 years) signify that research priorities established in the earlier post-diagnosis periods continue to influence research themes during this mid-term phase. These correlations demonstrate a lasting impact and the persistence of certain research directions initiated in the earlier years.

Even beyond 10 years post-diagnosis, the correlations remain relatively high (0.8911 with the first year, 0.8589 with 1–3 years, and 0.8291 with 4–10 years). This suggests that the research focus during the early years of post-diagnosis continues to influence and guide research priorities in the long term. The enduring correlation implies that certain research themes initiated shortly after diagnosis retain their relevance and continue to guide research endeavours even after a decade following the diagnosis.

Overall, this interpretation underscores the importance of understanding the trajectory of research priorities over time following a cancer diagnosis, showcasing how early research areas can have a lasting impact on long-term research strategies and directions.

#### Rank and Percentile

The table presents data on research priorities in the context of the time since cancer diagnosis. The data is divided into four categories: 1 year since diagnosis, 1–3 years since diagnosis, 4–10 years since diagnosis, and more than 10 years since diagnosis.

One noticeable trend in this data is that Measure ID 10, related to cancer research priorities, consistently maintains a top ranking across all four-time categories. This indicates a consistent focus on this particular research area regardless of how long it has been since the cancer diagnosis. It suggests that initiatives related to Measure ID 10, which may include early detection and screening efforts, remain of paramount importance throughout the journey of cancer patients. This consistency underscores the significance of early intervention in cancer care.

However, as the time since diagnosis increases, some shifts in research priorities become apparent. For example, Measures 30 and 14 are more highly ranked for individuals diagnosed 1–3 years ago compared to those diagnosed more than 10 years ago. This may indicate a greater emphasis on these measures during the earlier stages of post-diagnosis treatment and care. Conversely, Measure ID 4, which relates to novel therapies and treatment options, ranks higher among those diagnosed more than 10 years ago, suggesting a focus on long-term survivorship and alternative treatments (Table 5).

The data highlights the evolving needs and preferences of cancer patients based on the time since their diagnosis. For healthcare professionals and researchers, this information is invaluable in tailoring their efforts to cater to the specific concerns and priorities of individuals at different stages of their cancer journey. By understanding these variations, healthcare providers can offer more personalized care, and researchers can direct their resources toward areas that align with the evolving needs of cancer patients as they progress through their cancer experience. This insight underscores the importance of dynamic and adaptable cancer care and research strategies.

### 3.3. Treatment Received

The correlation table reveals the associations between different types of treatments administered to patients’ post-cancer diagnosis. Surgery, a foundational treatment in cancer care, exhibits a strong positive correlation with radiotherapy (r = 0.986), underlining their often-combined usage in treatment strategies. Additionally, surgery also correlates positively with chemotherapy (r = 0.988) and precision therapy (r = 0.902), emphasizing its integration into diverse treatment plans. Radiotherapy displays a strong positive correlation with both surgery (r = 0.986) and chemotherapy (r = 0.980), underscoring its pivotal role in various multidimensional treatment approaches. Precision therapy, a hallmark of personalized medicine, correlates strongly with chemotherapy (r = 0.896), shedding light on its potential synergies in tailored treatment regimens. Chemotherapy, a widely used treatment modality, showcases a strong positive correlation with surgery (r = 0.988) and precision therapy (r = 0.896), emphasizing its integral role and potential in combined treatment strategies. Biological therapies, an evolving frontier, exhibit a moderate to strong positive correlation with all other treatments, indicating their potential combined usage in diverse treatment plans. Hormone therapy, another vital treatment option, demonstrates a strong positive correlation with surgery (r = 0.960) and radiotherapy (r = 0.969), showcasing its frequent integration into these treatment approaches. Other therapies exhibit moderate positive correlations with various treatments, suggesting their supplementary role in cancer care and prompting further exploration for optimized treatment combinations (Table 6).

#### Rank and Percentile

The treatments are categorized into different modalities, including surgery, radiotherapy, precision therapy, chemotherapy, biological therapies, hormone therapy, and other therapies.

One noticeable pattern in this data is that for each treatment modality, there is a consistent top-ranking research priority. For example, Measure ID 14, which corresponds to precision therapy, consistently holds the top rank across all treatment modalities. This suggests that precision therapy, which involves targeting treatment based on an individual’s genetic makeup, is a high-priority research area regardless of the treatment method. It highlights the significance of personalized medicine and the quest for more effective and tailored treatments for cancer patients.

Additionally, Measure ID 10, associated with surgery, and Measure ID 30, related to radiotherapy, maintain high ranks across all treatment categories. This indicates that surgical interventions and radiotherapy remain critical areas of research, reflecting the importance of developing advanced techniques and technologies to improve patient outcomes.

There is also variation in research priorities between different treatments. For instance, Measure ID 11, related to other therapies, holds a lower rank compared to other treatment modalities. This suggests that research into alternative or complementary therapies may not be as prominent in the current landscape, emphasizing the need for more investigation in this area, especially considering patient interest in holistic treatment approaches.

Precision Therapy: Measure ID 14 consistently ranks at the top across all time frames with a 100% score. This demonstrates a strong research emphasis on precision therapy, a treatment approach that targets specific genetic factors in individual patients. The uniform high ranking reflects the importance of personalized medicine and the quest for more effective and tailored cancer treatments.

Chemotherapy: Measure ID 10 ranks consistently high across all time frames, indicating that chemotherapy remains a research priority in the field of cancer treatment. It’s vital to improve the effectiveness of chemotherapy, reduce side effects, and develop more targeted approaches, which is reflected in its rankings across different time frames.

Biological Therapies: Measure ID 14 holds a consistent top rank with a 100% score in the 1-year and 4–10-year categories, indicating its sustained research importance. However, its ranking drops slightly in the 1–3 year category. This suggests a focus on short-term research priorities in biological therapies and a potential shift in emphasis over the years.

Hormone Therapy: Measure ID 10 ranks high across all time frames. This indicates ongoing research efforts to enhance hormone therapy’s effectiveness, especially in the first year of diagnosis. The consistent ranking suggests that hormone therapy remains a cornerstone in cancer treatment.

Other Therapies: Measure ID 14 holds a high rank in the 1-year and 4–10-year categories, but its ranking drops in the 1–3-year category. This may imply that research priorities for other therapies differ depending on the time frame. It’s important to explore what these “other therapies” entail and adapt research accordingly (Table 7).

## 4. Discussion

### 4.1. Demographic Analysis and Research Priorities

The analysis offers valuable insights into the relationship between demographic factors and cancer research priorities. 

Age Groups: The analysis reveals an interesting trend in the correlation between age groups and their cancer research priorities. The data suggests that there is a significant alignment in research focus between individuals aged 26–44 and 45–69. This high correlation (approximately 0.979) among those aged 45–69 is noteworthy. It may be attributed to several factors, including the prevalence of cancer in this age group and the greater need for research to address their specific concerns. This finding underscores the importance of tailoring cancer research efforts to address the needs of the middle-aged and older population. 

Age Group Divergence: In contrast, the analysis shows that individuals over 70 years have a lower correlation (approximately 0.416) with younger age groups. This suggests a divergence in research priorities among the elderly compared to younger individuals. This divergence could be due to different cancer types, comorbidities, and treatment preferences among the elderly. As the aging population continues to grow, this finding emphasizes the importance of addressing the unique needs of older individuals in cancer research. 

For healthcare providers, understanding the treatment outcomes that older cancer patients prioritize is crucial. This knowledge allows them to customize the information and treatment recommendations for these patients, recognizing that their priorities may differ from those of younger patients. In the analysis of 28 studies involving 4374 patients, conducted by Seghers et al., they found that only six of these studies focused primarily on older patients. Despite quality of life being considered in only half of the studies, it was the most commonly rated as the highest or second-highest priority (79%). This was followed by overall survival (67%), progression- and disease-free survival (56%), the absence of severe or persistent treatment side effects (54%), treatment response (50%), and the absence of transient short-term side effects (16%). These findings can inform shared decision-making, enabling healthcare professionals to better tailor their information and treatment recommendations to each patient’s unique preferences and needs [31].

Gender: The correlation analysis between genders (approximately 0.769) reveals a high degree of alignment in cancer research priorities. Despite potential gender-specific factors in cancer incidence and treatment, both males and females share similar priorities in certain areas of cancer research. This alignment may reflect the common goal of reducing the overall burden of cancer and suggests that many aspects of cancer research benefit all genders equally.

Age-Gender Relationships: The correlation between gender and age groups shows interesting variations. It appears that females in the 16–25 age group exhibit a stronger correlation (approximately 0.742) with this age category than males (approximately 0.442). This could be due to a higher incidence of specific cancers among young females or varying research interests. As age advances, the alignment between gender and age decreases, which implies that the gender-specific influence on research priorities becomes less pronounced as individuals grow older. This finding highlights the importance of understanding age-gender dynamics when designing targeted interventions and research strategies.

In conclusion, the analysis of correlations among age groups, gender, and age-gender relationships provides a comprehensive view of the nuances in cancer research priorities within different demographic segments. It emphasizes the need for a multifaceted approach to cancer research that considers the specific needs of various age groups and genders. As the population continues to age and our understanding of cancer evolves, research priorities should adapt to ensure that the most pressing concerns are addressed effectively.

Overall, the examination of age groups reveals a noteworthy correlation between individuals aged 26–44 and 45–69, emphasizing the importance of tailoring cancer research efforts to address the needs of the middle-aged and older population. However, a divergence is observed among individuals over 70 years, emphasizing the necessity of addressing the unique needs of older individuals in cancer research. This supports the introduction’s emphasis on assessing healthcare needs in different age groups. Further, the need for healthcare providers to understand treatment outcomes prioritized by older cancer patients, reinforcing the importance of tailoring information and recommendations.

### 4.2. Time First Diagnosed and Research Priorities

The analysis of correlations between time since cancer diagnosis and research priorities offers valuable insights into how research themes evolve and persist over time. The following discussion explores the implications of these correlations:

1. Early-Stage Research Persistence: The high correlation of 1.000 during the first year after diagnosis indicates a strong and immediate alignment in research priorities during this critical period. This makes sense, as the initial phase following a cancer diagnosis is marked by intensive research to address immediate concerns, such as treatment options and disease management. This correlation underscores the significance of early-stage research, suggesting that the groundwork laid in the first year serves as a foundation for later research efforts.
2. Consistency in Short to Mid-Term Priorities: The continued high correlation (0.9318) during the 1–3 year period post-diagnosis indicates that research priorities remain notably aligned during this short to mid-term phase. This suggests that the research themes established in the first year continue to guide research efforts into the subsequent years. It reflects the need for a consistent and coherent approach to addressing the evolving needs of patients in the years immediately following their diagnosis.
3. Mid-Term Research Influence: As patients move into the 4–10-year timeframe after diagnosis, the correlations of 0.8526 (with the first year) and 0.8952 (with 1–3 years) show that research priorities in the earlier post-diagnosis periods significantly influence research directions during this mid-term phase. This reveals the lasting impact of research initiatives from the earlier years. It’s indicative of the need for sustained focus on specific research areas to ensure that they have a meaningful impact on the lives of cancer survivors.
4. Long-Term Research Continuity: Even beyond 10 years post-diagnosis, the correlations remain relatively high (0.8911 with the first year, 0.8589 with 1–3 years, and 0.8291 with 4–10 years). This implies that research themes initiated shortly after diagnosis continue to shape research priorities in the long term. The persistence of these correlations emphasizes that certain research directions maintain their relevance and continue to guide research endeavors over a decade following the diagnosis. It suggests that long-term studies should take into account the groundwork laid during the initial years to ensure a consistent and effective approach to cancer research.

In summary, this analysis highlights the importance of a structured and continuous approach to cancer research. It demonstrates the enduring influence of early-stage research priorities, underlining the need for ongoing support and adaptation to effectively address the evolving needs of cancer patients over time. By understanding how research priorities evolve and persist, researchers can better tailor their efforts to ensure long-term, meaningful outcomes in the fight against cancer. Patient and public partnerships in medical research have gained significant importance, seen as both an ethical imperative and vital for enhancing the quality, safety, value, and sustainability of healthcare systems and research. The absence of patient involvement in biomedical research is recognized as a source of resource wastage [32]. Community engagement is a vital component in the efforts to tackle cancer disparities. It actively engages communities affected by cancer in the creation, execution, and assessment of initiatives and interventions. This approach acknowledges that communities possess expertise regarding their own experiences, requirements, and choices, and it empowers them to take an active role in decision-making processes [33].

### 4.3. Treatment Received Analysis and Research Priorities

The analysis of correlations between different types of cancer treatments administered to patients provides valuable insights into the patterns and associations among these therapies.

1. Surgery’s Central Role: Surgery, often the initial and foundational treatment in cancer care, exhibits strong positive correlations with various other treatment modalities. It shows particularly robust associations with radiotherapy (r = 0.986) and chemotherapy (r = 0.988), suggesting that surgery is often combined with these treatments in comprehensive cancer management. This underlines the central role of surgery in the multidimensional approach to cancer treatment. In the field of oncology, the multidisciplinary approach came into prominence during the mid-1980s. This shift occurred when it was established that combining chemotherapy with radiotherapy and/or surgery led to enhanced survival rates. When assessing the results of individual treatment methods like surgery or radiotherapy, no distinctions were observed between younger and older patients [34].
2. Radiotherapy’s Integration: Radiotherapy is another pivotal treatment in cancer care, and the analysis reveals strong positive correlations with both surgery (r = 0.986) and chemotherapy (r = 0.980). These strong correlations emphasize the common integration of radiotherapy into multidisciplinary treatment plans. The alignment between surgery and radiotherapy is especially notable, as these treatments are often used in sequence to maximize effectiveness. Technological advancements and progress in radiobiological research have enabled the delivery of increasingly personalized radiation treatments in a more efficient and streamlined manner. This improvement enhances the effectiveness, safety, and availability of radiation therapy. While these changes contribute to the enhancement of cancer care quality, they also significantly raise the complexity of decision-making, thereby posing a challenge to the delivery of high-quality yet affordable cancer care [35].
3. Chemotherapy’s Integral Role: Chemotherapy, a widely used and versatile treatment modality, demonstrates strong positive correlations with surgery (r = 0.988) and precision therapy (r = 0.896). These strong associations highlight the integral role of chemotherapy in combined treatment strategies, where it complements surgical and precision therapy approaches.
4. Precision Therapy Synergy: Precision therapy, a hallmark of personalized medicine, correlates strongly with chemotherapy (r = 0.896), indicating its potential synergies in tailored treatment regimens. This suggests that precision therapy is often considered alongside chemotherapy to provide a more personalized and effective approach to cancer treatment. The diversity of diseases within the realm of cancer, marked by variations in genetic causes and protein expressions from one patient to another, renders conventional treatments like chemotherapy and radiation effective for only a specific subset of individuals. This inherent heterogeneity in cancer has given rise to the burgeoning field of precision and personalized medicine (PPM). Current endeavours are focused on gathering PPM data to better understand the molecular distinctions between different tumours [36].
5. Biological Therapies’ Versatility: Biological therapies, an evolving frontier in cancer treatment, exhibit moderate to strong positive correlations with all other treatments. This suggests their potential combined usage in diverse treatment plans. Biological therapies, being more targeted, are often integrated with other modalities to enhance treatment efficacy.
6. Hormone Therapy’s Complementarity: Hormone therapy, which is a vital treatment option for hormone-driven cancers, demonstrates strong positive correlations with surgery (r = 0.960) and radiotherapy (r = 0.969). These correlations underscore the frequent integration of hormone therapy into treatment approaches involving surgery and radiotherapy, especially in cases where hormone receptors play a significant role in cancer development.
7. Supplementary Role of Other Therapies: Other therapies exhibit moderate positive correlations with various treatments, suggesting their supplementary role in cancer care. These therapies likely complement primary treatment modalities and offer additional options for patients, demonstrating the multidimensional nature of cancer treatment.

In summary, the correlations between different cancer treatment modalities highlight the multidisciplinary and integrative approach to cancer care. These findings underscore the importance of tailoring treatment plans to individual patient needs and the potential benefits of combining various treatment modalities to achieve the best possible outcomes in the fight against cancer. Additionally, this analysis provides insights into the evolving landscape of cancer treatment and the increasing focus on personalized and targeted therapies. The priorities in cancer medicine emphasizes the necessity of a multi-disciplinary, precision-oriented approach, integrating various therapies, and adapting to the evolving landscape of oncology. Key priorities include optimizing systemic and locoregional treatments, leveraging precision oncology with biomarkers and technology, and fostering international collaboration. In radiation oncology, the focus is on refining treatment strategies, understanding the role of particle therapy, and incorporating AI. The EORTC-ESTRO Radiation Infrastructure for Europe (E2RADIatE) is highlighted as a valuable tool for prospective clinical trials. The sustainability of such platforms is a major concern, calling for support from governments and funding agencies to benefit patients and society by advancing therapeutic strategies that truly make a difference [37,38,39,40].

### 4.4. Bias Arising from Methods of Recruitment

Sampling Bias:

- The survey included participants from 30 European countries, but the sample may not be fully representative of the diverse population in each country.
- Certain demographic groups may be underrepresented, leading to potential sampling bias.

Self-Reported Data:

- The study relies on self-reported data from survey participants, which may be subject to recall bias and social desirability bias.
- Participants might provide responses they perceive as socially acceptable, potentially affecting the accuracy of the findings.

Generalizability:

- Findings from this study may not be fully generalizable to populations outside the age range of 16 to 70+ or to regions/countries not included in the study.

Cross-Sectional Design:

- The study’s cross-sectional design provides a snapshot of attitudes and perspectives at a specific point in time. Longitudinal data would offer a more comprehensive understanding of how these perspectives evolve over time.

Limited Scope of Cancer Types:

- The study focused on specific cancer types (breast cancer, lung cancer, prostate cancer, colon, and other gastrointestinal cancers), potentially overlooking perspectives on less common cancers.

Influence of Health Systems:

- The study may not fully account for variations in healthcare systems across different European countries, which could influence perspectives on cancer care.

### 4.5. Alignment with EU Mission: Cancer

The study aligns closely with the EU Mission’s research priorities and funding opportunities in cancer research and innovation. By analyzing correlations among age groups, genders, and age-gender relationships, the study provides insights into cancer research priorities across different demographic segments, supporting the Mission’s objectives of understanding cancer, prevention, early detection, diagnosis, treatment, and improving quality of life for patients and their families. Moreover, the analysis of treatment received and research priorities sheds light on the multidisciplinary and integrative approach to cancer care, which resonates with the Mission’s emphasis on optimizing treatment strategies and leveraging precision oncology. The study’s focus on tailoring cancer research efforts to address the specific needs of different age groups and genders also aligns with the Mission’s goal of providing a better understanding of cancer and improving cancer patients’ quality of life. The mention of funding opportunities under the Horizon Europe Programme, EU4Health Programme, Digital Europe Programme, Euratom Programme, and Interregional Innovation Investments funding instrument underscores the potential for the study’s findings to contribute to the broader research and innovation landscape in cancer control.

Overall, the study’s findings and methodologies are in line with the EU Mission’s objectives and provide valuable insights that can inform future research, innovation, and policy initiatives in cancer care.

## 5. Conclusions

Data on cancer research priorities paints a nuanced picture of what various demographic groups believe should be the focus of cancer research. The consensus among different age groups on certain research areas underscores their critical importance. At the same time, recognizing gender-based differences can lead to more targeted research and healthcare interventions, demonstrating the importance of developing patient and public involvement (PPI) in healthcare and research in Europe. The insights from this study provide a roadmap for the allocation of resources and funding, ensuring that cancer research is both comprehensive and responsive to the diverse needs of the population. Researchers, policymakers, and healthcare professionals should take these priorities into account when developing strategies to combat this complex and challenging disease.

Based on the findings and insights from the study, several policy recommendations could be considered to improve cancer research, prevention, and healthcare across different age groups in Europe:

- Tailored Public Health Campaigns:

Develop and implement age-specific public health campaigns to raise awareness about cancer prevention measures tailored to the perceptions and preferences of different age cohorts.

- Age-Targeted Early Detection Programs:

Design and implement targeted early detection programs that take into account the prevalent cancers within specific age groups, aiming for early diagnosis and improved treatment outcomes.

- Patient-Centric Treatment Approaches:

Encourage healthcare providers to consider age-specific factors in treatment decisions, ensuring that cancer treatment approaches are tailored to the unique needs and preferences of different age cohorts.

- Inclusive Cancer Research Prioritization:

Incorporate age-related concerns and priorities into the prioritization of cancer research efforts, ensuring that research investments address the distinct challenges faced by individuals within different age ranges.

- Resource Allocation Based on Age-Specific Analysis:

Guide policymakers in efficiently allocating healthcare resources based on age-specific analysis, allowing for effective provision of healthcare that addresses the varying importance of cancer treatment measures within distinct age categories.

- Support for Cancer Caregivers:

Develop policies to provide support and resources for caregivers, recognizing the vital role they play in the care of cancer patients across different age groups.

- Enhanced Health Education Programs:

Strengthen health education programs targeted at specific age groups to improve understanding and awareness of cancer-related issues, emphasizing prevention, early detection, and available treatment options.

- Cross-Country Collaboration:

Encourage collaboration and information sharing among European countries to facilitate a comprehensive understanding of regional and country-specific variations in cancer perspectives, enabling the development of more targeted interventions.

- Patient Involvement in Decision-Making:

Promote policies that encourage the active involvement of patients and the public in decision-making processes related to cancer research priorities, treatment guidelines, and healthcare policies.

## Figures and Tables

**Table 1:** Pillars and measures identified during the analysis.

Measure ID	Pillar ID	Measure Name	Measure Description
1	1	Gut Microbiome and Dietary Impact	The last decade has brought us a greater understanding of the impact of our ‘diet’ on intestinal ‘microbiota’ (gut bacteria), and how changes in the ‘microbiota’ are associated with our health (cancer promotion and prevention).
2	1	Metabolic Health and Physical Activity Influence	Studies have shown that lifestyle behaviours may impact metabolism and cancer risk.
3	1	Prolonged Inflammatory Responses	Studies have shown that inflammation that becomes chronic or lasts for too long is often associated with the development and progression of cancer.
4	1	Environmental Carcinogenic Factors	Studies have shown that some environmental factors, called carcinogens, increase the risk of developing cancer.
5	2	Cancer Risk Reduction Strategies	by the immune system and chemo treatments by using for instance vaccines, such as HPV vaccines, or preventive drugs for certain cancer types.
6	2	Genetic and Epigenetic Cancer Influences	Studies have shown that cancers develop due to the accumulation of genetic (changes in the DNA sequence, some of which may be inherited) and epigenetic (changes not affecting the DNA sequence but its activity, that are non-inherited) alterations.
7	2	Pre-Tumor Progression Phases	The development of cancer is a multistep process in which normal cells gradually become malignant through progressive accumulation of molecular alterations.
8	2	Initial Cancer Development Phases	Cancer is a disease caused when cells divide uncontrollably and cooperate with other cells in their local environment which fosters tumour progression.
9	2	Hematological Biomarkers for Early Detection	Specific blood tests are designed to identify tumour (bio)markers that may be found in the blood when some cancers are present before showing symptoms or being detected through conventional imaging approaches.
10	2	Advanced Early Cancer Diagnostic Technologies	Numerous cancer-associated deaths occur from cancers for which we do not screen. To overcome this, new scalable and cost-effective technologies are developed to allow for the detection and diagnosis of cancers at an earlier stage when these are more responsive to treatments.
11	2	Tailored Cancer Risk Management and Early Screening	Everybody does not have the same risk of developing cancer. Careful analysis of individual risk factors to adapt prevention and systematic screening to the risk level would increase the rate of early diagnosis
12	2	Hematological Assays for Treatment Responsiveness and Resistance	In the past two decades, specific tests have been developed to customize the treatment plan for a cancer patient according to the sensitivity and resistance patterns that can be monitored by analyzing the patient’s blood.
13	3	Cancer Cell Biology and Immune Microenvironment	Studies have shown that not all cancer cells are created equal, and they can remodel the cells around them. There are intrinsic differences in the proliferative and invasive capacity of cancer cells within the same patient, and immune cells in their environment also acquire specific properties.
14	3	Innovative Anti-Cancer Therapies and Drug Delivery Methods	The development of more specific anti-cancer drugs, new types of biological and immune-mediated therapies, a combination of therapies with diverse mechanisms of action, and advanced drug delivery systems to target cancer cells more specifically, have the potential to improve cancer treatment for patients and reduce long-term effects.
15	3	Hereditary Factors and Epigenetic Mechanisms in Pediatric Oncology	The contribution of non-genetic factors and the influence of the tissue environment remains poorly understood.
16	3	Oncogenesis and Growth Phases	The causes of the molecular changes during development that lead to cancer in children are mostly unknown.
17	3	Therapeutic Approaches for Pediatric Cancers	What is effective for an adult with cancer might not work for a pediatric cancer patient. Therefore, specific strategies to treat pediatric and adolescent cancer patients are needed.
18	3	Immunological Aspects in Pediatric Cancer	The immune system of children and adolescents is different from that of an adult, and the efficacy of immunotherapy might vary depending on the age of the patient and needs to be understood better.
19	3	Maternal Factors and Pediatric Cancer Association	Epidemiological studies have suggested an association between maternal risk factors or exposure to carcinogens during pregnancy, with pediatric cancer incidence. However, the precise factors and mechanisms involved remain unexplored.
20	4	Aging Factors and Cancer Susceptibility	The incidence of most cancers increases with age as, for most adults, age is associated with chronic conditions, decreased efficacy of the immune system, cumulative exposure to risk factors (carcinogens), and tissue ageing with cell senescence, which is causally associated with cancer.
21	4	Cellular Senescence in Cancer Biology	Aging is a complex phenomenon caused by the time-dependent loss of physiological organism functions including those that protect from cancer development.
22	4	Ageing and Carcinogenesis Relationship	Studies have shown that mechanisms of ageing are also found to occur in carcinogenesis. There is a need to better understand what ageing and cancer development share and where the two processes diverge.
23	4	Aging Impact on Cancer Treatments	Various studies support the hypothesis that cancer and/or cancer treatment are associated with accelerated biological ageing. This is a key determinant of survivorship along with the long-term impact of cancer therapy on the biological ageing of an individual.
24	5	Adverse Events and Concurrent Medical Conditions in Cancer	In older patients affected by cancer, it is key to consider not only the characteristics of the tumour but also pursue an integral geriatric assessment to systematically investigate factors that determine the well-being of patients. In this context, research suggests that we may be able to measure a biological age, which will be more precise than civil age to guide therapeutic choices when treating a cancer.
25	5	Treatment-Related Secondary Neoplasms	Though it happens infrequently, patients may develop a secondary cancer as a result of the treatment received to treat the primary cancer.
26	5	Persistent Immunological Consequences of Treatment	The effects of some cancer treatments can compromise some properties of the immune system, rendering patients vulnerable to viral and bacterial infections or causing autoimmune conditions.
27	5	Reproductive Health Impact due to Cancer and Treatment	Cancer and its treatment can adversely impact reproductive function in both women and men. The effects of cancer treatment may lead to transient or permanent loss of fertility, sexual desire, and function.
28	5	Cardiovascular, Respiratory, and Hormonal Health Impact due to Treatment	Both chemotherapy and radiation therapy to the chest can cause problems in the heart and lungs leading to potential cardiovascular or respiratory conditions that may be temporary or long-lasting.
29	5	Neurological Consequences of Cancer Treatments	Chemotherapy and radiation therapy can cause long-term side effects on the brain, spinal cord, and nerves, and sometimes enhance pain sensitivity.
30	5	Holistic Care for Cancer Survivors	For cancer survivors who are no longer in active treatment, their care needs include surveillance for recurrence, screening for the development of subsequent primary cancers, monitoring and intervention for the long-term and late physical and psychological effects of cancer and its treatment, management of comorbid medical conditions, as well as routine preventive and primary care.
31	6	Data Generation in Oncological Research	The development of data that may guide more precise therapeutic choices and generate more efficacy in treating cancer patients.
32	6	Data Utilization for Informed Oncological Decision-making	Data whose analysis can inform on disease precise diagnosis, its heterogeneity, the existence of constitutive predisposing factors, and the ability of the patient to support and favourably respond to a given therapy.
33	6	Data Collection and Analysis in Oncology	With the tools of data sciences, researchers can collect and analyze data to identify common mechanisms in a large series of patients with similar diseases. With data sciences, the higher the number of patients analyzed, the more precise the analysis.
34	6	Data Quality Assurance in Oncological Studies	The efficacy of data sciences requires data standardization and interoperability to be re-used by multiple teams asking complementary questions.
35	6	Regulated Sharing of Patient Data for Oncology Research	Patient data sharing requires strict regulation to protect privacy (anonymization). While such regulation is mandatory, it must also be organized in a manner that favours rather than prevents patient data sharing at the European level to support cancer research.

**Table 2:** Correlations Between Gender and Age.

Correlation	16–25 Years Old	26–44 Years Old	45–69 Years Old	Over 70 Years Old	Male	Female
16–25 years old	1	0.7379438335	0.6675768386	0.4160364372	0.4418178557	0.7415742331
26–44 years old	0.7379438335	1	0.9347960985	0.6613814037	0.7646749705	0.9711350806
45–69 years old	0.6675768386	0.9347960985	1	0.7187687319	0.8558082488	0.9791431967
over 70 years old	0.4160364372	0.6613814037	0.7187687319	1	0.7531487128	0.7155098324
Male	0.4418178557	0.7646749705	0.8558082488	0.7531487128	1	0.7692347738
Female	0.7415742331	0.9711350806	0.9791431967	0.7155098324	0.7692347738	1

**Table 3:** Rank and Percentile—The data provide insights into how specific cancer research measures rank across different demographics, age and gender.

Measure ID	16–25	Rank	Percent	Measure ID	26–44	Rank	Percent	Measure ID	45–69	Rank	Percent	Measure ID	Over 70	Rank	Percent	Measure ID	Male	Rank	Percent	Measure ID	Female	Rank	Percent
11	76.0	1	100.00%	17	65.8	1	100.00%	10	65.2	1	100.00%	12	55.9	1	100.00%	10	60.4	1	100.00%	10	65.1	1	100.00%
9	72.0	2	85.20%	14	65.3	2	97.00%	30	60.7	2	97.00%	4	52.9	2	94.10%	9	56.3	2	97.00%	14	62.9	2	97.00%
10	72.0	2	85.20%	10	64.8	3	94.10%	9	58.8	3	91.10%	10	52.9	2	94.10%	35	55.6	3	94.10%	17	62.4	3	94.10%
16	72.0	2	85.20%	9	61.8	4	91.10%	14	58.8	3	91.10%	34	51.5	4	91.10%	32	53.5	4	91.10%	30	60.2	4	91.10%
17	72.0	2	85.20%	11	59.8	5	88.20%	17	58.2	5	88.20%	14	48.5	5	82.30%	4	52.8	5	85.20%	9	59.6	5	88.20%
28	72.0	2	85.20%	18	59.3	6	85.20%	18	56.2	6	85.20%	32	48.5	5	82.30%	33	52.8	5	85.20%	18	58.3	6	85.20%
4	48.0	30	11.70%	1	33.7	30	14.70%	2	34.6	30	14.70%	24	32.4	27	14.70%	20	27.1	30	14.70%	24	36.3	30	14.70%
31	48.0	30	11.70%	24	33.2	31	11.70%	23	32.9	31	11.70%	6	30.9	31	11.70%	22	26.4	31	11.70%	23	35.2	31	11.70%
22	44.0	32	8.80%	23	32.2	32	8.80%	20	31.9	32	8.80%	27	29.4	32	8.80%	2	25.7	32	5.80%	22	33.4	32	8.80%
20	40.0	33	2.90%	22	31.2	33	5.80%	1	31.3	33	2.90%	2	27.9	33	5.80%	27	25.7	32	5.80%	20	32.5	33	5.80%
21	40.0	33	2.90%	21	30.2	34	2.90%	22	31.3	33	2.90%	3	20.6	34	2.90%	1	25.0	34	2.90%	1	31.7	34	2.90%
1	36.0	35	0.00%	20	29.1	35	0.00%	21	28.6	35	0.00%	1	11.8	35	0.00%	21	24.3	35	0.00%	21	30.9	35	0.00%
* 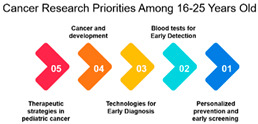 *								* 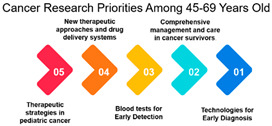 *								* 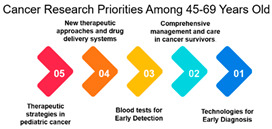 *							
* 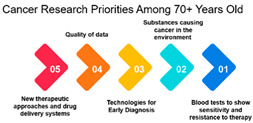 *								* 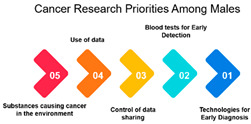 *								* 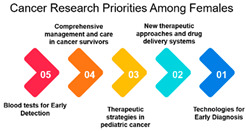 *							

**Table 4:** The correlation table displays the trends in cancer research priorities based on the time intervals since the patients’ diagnosis.

Correlation	1 yr	1–3 yr	4–10 yr	More than 10 yr
1 yr	1	0.931755426	0.8526427769	0.8910662593
1–3 yr	0.931755426	1	0.8951936613	0.8589079186
4–10 yr	0.8526427769	0.8951936613	1	0.8290505511
more than 10 yr	0.8910662593	0.8589079186	0.8290505511	1

**Table 5:** Rank and Percentile—Data on research priorities in the context of the time since cancer diagnosis.

Measure ID	1 yr	Rank	Percent	Measure ID	1–3 yr	Rank	Percent	Measure ID	4–10 yr	Rank	Percent	Measure ID	More than 10 yr	Rank	Percent
10	58.0	1	100.00%	10	67.8	1	97.00%	10	61.2	1	100.00%	10	64.4	1	100.00%
30	55.7	2	97.00%	14	67.8	1	97.00%	9	60.3	2	97.00%	31	60.0	2	97.00%
14	55.0	3	94.10%	17	62.5	3	94.10%	30	55.5	3	94.10%	4	59.3	3	88.20%
32	53.4	4	91.10%	9	59.1	4	91.10%	11	53.6	4	88.20%	30	59.3	3	88.20%
9	52.7	5	85.20%	12	57.2	5	85.20%	14	53.6	4	88.20%	35	59.3	3	88.20%
17	52.7	5	85.20%	30	57.2	5	85.20%	17	52.2	6	85.20%	9	57.8	6	85.20%
3	32.8	28	14.70%	3	35.6	28	20.50%	2	36.8	28	20.50%	27	40.0	28	20.50%
21	32.8	28	14.70%	22	35.1	29	17.60%	24	34.4	29	14.70%	23	36.3	29	17.60%
24	32.8	28	14.70%	23	34.6	30	14.70%	27	34.4	29	14.70%	2	35.6	30	14.70%
20	31.3	31	8.80%	27	34.1	31	11.70%	23	33.0	31	11.70%	22	32.6	31	11.70%
22	31.3	31	8.80%	24	33.7	32	8.80%	1	30.6	32	8.80%	3	31.9	32	5.80%
1	30.5	33	5.80%	1	33.2	33	5.80%	20	29.2	33	5.80%	20	31.9	32	5.80%
23	29.0	34	2.90%	20	32.2	34	2.90%	22	27.3	34	2.90%	21	26.7	34	2.90%
2	28.2	35	0.00%	21	31.7	35	0.00%	21	26.3	35	0.00%	1	23.7	35	0.00%
* 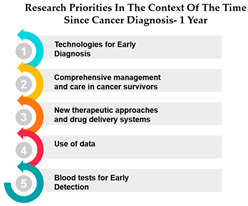 *								* 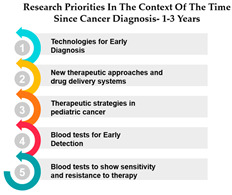 *							
* 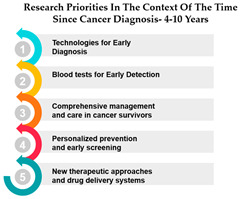 *								* 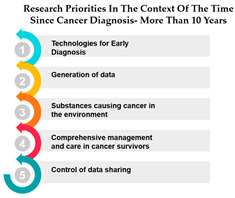 *							

**Table 6:** The correlation table shows the associations between different types of treatments administered to patients post-cancer diagnosis.

Correlation	Surgery	Radiotherapy	Precision Therapy	Chemotherapy	Biological Therapies	Hormone Therapy	Other Therapies
Surgery	1	0.9857459184	0.9019100331	0.9876912185	0.9193993214	0.9599388983	0.8657818333
Radiotherapy	0.9857459184	1	0.8788912483	0.9797086469	0.9058054009	0.9689716625	0.8347353961
Precision Therapy	0.9019100331	0.8788912483	1	0.8968722281	0.8508449381	0.8820733325	0.9041920792
Chemotherapy	0.9876912185	0.9797086469	0.8968722281	1	0.9437853263	0.9554271538	0.8658437144
Biological Therapies	0.9193993214	0.9058054009	0.8508449381	0.9437853263	1	0.8969734276	0.8307120919
Hormone Therapy	0.9599388983	0.9689716625	0.8820733325	0.9554271538	0.8969734276	1	0.8264734934
Other Therapies	0.8657818333	0.8347353961	0.9041920792	0.8658437144	0.8307120919	0.8264734934	1

**Table 7:** Rank and Percentile—Data on research priorities in the context of the different therapies.

Measure ID	Surgery	Rank	Percent	Measure ID	Radiotherapy	Rank	Percent	Measure ID	Precision Therapy	Rank	Percent	Measure ID	Chemotherapy	Rank	Percent	Measure ID	Biological Therapies	Rank	Percent	Measure ID	Hormone Therapy	Rank	Percent	Measure ID	Other Therapies	Rank	Percent
10	64.0	1	100.00%	10	63.2	1	100.00%	14	75.0	1	100.00%	10	64.7	1	100.00%	14	66.1	1	100.00%	10	61.9	1	100.00%	14	82.9	1	100.00%
9	59.2	2	97.00%	30	59.6	2	97.00%	10	73.4	2	97.00%	14	60.2	2	97.00%	10	65.3	2	94.10%	17	60.1	2	97.00%	9	73.2	2	97.00%
30	58.6	3	94.10%	14	58.2	3	94.10%	9	65.6	3	94.10%	30	58.8	3	94.10%	32	65.3	2	94.10%	11	59.6	3	91.10%	10	68.3	3	88.20%
14	56.7	4	88.20%	9	57.9	4	88.20%	11	64.1	4	88.20%	9	58.5	4	91.10%	17	64.5	4	88.20%	14	59.6	3	91.10%	11	68.3	3	88.20%
17	56.7	4	88.20%	17	57.9	4	88.20%	17	64.1	4	88.20%	17	56.6	5	88.20%	31	64.5	4	88.20%	30	57.8	5	88.20%	17	68.3	3	88.20%
23	34.2	31	11.70%	23	35.7	31	11.70%	19	32.8	31	2.90%	23	34.4	31	11.70%	2	36.4	31	11.70%	20	34.4	31	11.70%	3	36.6	30	11.70%
20	32.2	32	8.80%	22	34.0	32	8.80%	20	32.8	31	2.90%	20	33.4	32	8.80%	21	32.2	32	8.80%	23	33.9	32	8.80%	23	34.1	32	5.80%
22	31.6	33	5.80%	20	32.9	33	5.80%	21	32.8	31	2.90%	21	32.0	33	5.80%	22	29.8	33	5.80%	1	33.5	33	2.90%	27	34.1	32	5.80%
1	31.4	34	2.90%	21	30.6	34	2.90%	24	32.8	31	2.90%	22	31.8	34	2.90%	1	28.1	34	0.00%	22	33.5	33	2.90%	20	31.7	34	2.90%
21	29.4	35	0.00%	1	28.4	35	0.00%	27	29.7	35	0.00%	1	27.7	35	0.00%	23	28.1	34	0.00%	21	33.0	35	0.00%	21	26.8	35	0.00%
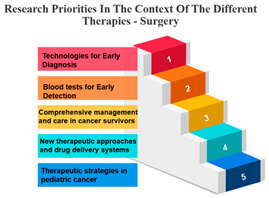									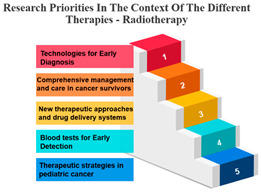										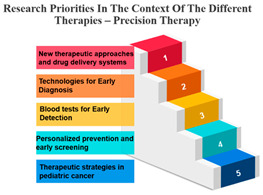								
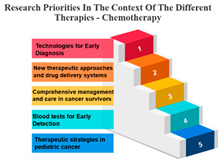							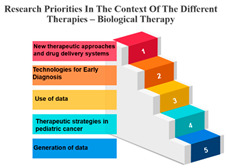							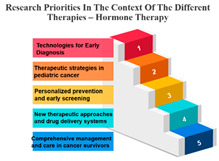							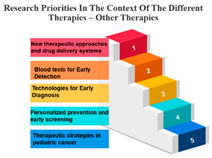						

## Data Availability

All data generated or analyzed during this study are included in this published article.

## Supplementary Materials

The following are available online at https://www.mdpi.com/article/10.3390/curroncol31040139/s1. Questionnaire.
