# Supplementary material for: Demographic Analysis of Cancer Research Priorities and Treatment Correlations
Source: Curr Oncol. 2024 Mar 29;31(4):1839–64. doi: 10.3390/curroncol31040139 (PMC11048756; doi:10.3390/curroncol31040139)
Supplement: Supplementary file 1 [file curroncol-31-00139-s001.zip › curroncol-2889198-supplementary.pdf]

Survey questions to patients:

Demographics and other

1. What is your gender?
- Male
- Female
- I prefer not to say

2. What is your age?
- 16 - 25
- 26 - 44
- 45 – 69
- 70 and over

3. What is your highest level of education?
- None of the basic education
- High school
- University degree
- Postgraduate studies
- I prefer not to say

4. What country are you currently living in?

[Drop-down field with all European countries]

5. In what type of area do you live?
- Rural
- Suburban
- Urban

6. What is your current working status?
- Employed
- Self-employed
- Unemployed and able to work
- Unemployed and not able to work
- Retired

- Student
- Housework
- Other

7. Has any of your close family ever been affected by cancer?
- Yes
- No
- Not sure

8. Which of the following categories best describes you [Allow multiple]
- I am an adult cancer patient
- I am an adult cancer survivor
- I am a pediatric cancer patient
- I am a pediatric cancer survivor
- I am a caregiver for an adult cancer patient
- I am a parent/caregiver for a pediatric cancer patient
- None of the above/not directly affected by cancer

9. What type(s) of cancer did you or do you currently have or the person you provide care for had or have? [Allow multiple]
- Breast cancer
- Lung cancer
- Colon cancer
- Other gastrointestinal cancer (e.g. stomach cancer or esophagus cancer)
- Prostate cancer
- Kidney cancer
- Other urogenital cancer (e.g. bladder cancer, testicle cancer)
- Gynaecological cancer (e.g. endometrial cancer, ovarian cancer)
- Melanoma (skin cancer)
- Leukemia
- Lymphoma
- Myeloproliferative disorder
- Other

10. When were you or the person you provide care for first diagnosed with cancer? [Single select]

- Less than 1 year
- 1-3 years
- 3-10 years
- more than 10 years

11. How were you or the person you provide care for diagnosed with cancer? [Allow multiple]

- Physical exam
- Blood and urine tests
- Imaging tests
- Biopsy
- Other
- I don’t know/I am not sure

12. What treatment have you or the person you provide care for received since diagnosis? [Allow multiple]

- Surgery
- Radiotherapy
- Chemotherapy
- Biological therapies (i.e. immunotherapy)
- Precision / targeted therapy
- Hormone therapy
- Other
- I don’t know/I am not sure

13. Which of these cancer research areas do you find important for the EU Commission to prioritize when it comes to new policies, budgets, and resources?
Scale from 1 to 5 (1 = ‘Not important at all’; 2 = ‘Slightly important’; 3 = ‘Obviously important’; 4 = ‘Strongly important’; 5 = ‘Absolutely important’) [Integer 1-5]. In case you are not aware of the item or not sure or don’t want to answer, please select ‘I don’t know’.

- Cancer Prevention
- Screening & Early Diagnosis

- Sensitivity and Resistance to therapy
- Pediatric Cancer
- Cancer and Ageing
- Survivorship & quality of life

14. Which of the following topics do you find the most important to prioritize within the area chosen?

CANCER PREVENTION

Cancer prevention is an action taken to lower the risk of getting cancer. This can include maintaining a healthy lifestyle, avoiding exposure to known cancer-causing substances, and taking medicines or vaccines that can prevent cancer from developing. Therefore, scientists are studying 'risk factors' (factors increasing the chance of developing cancer) and 'protective factors' (factors decreasing the chance of developing cancer) to find ways to prevent new cancers from initiating.

In this context, please rate the following topics on your perceived significance for further research in terms of their potential impact on cancer risk. Scale from 1 to 5 (1 = 'Not important at all'; 2 = 'Slightly important'; 3 = 'Obviously important'; 4 = 'Strongly important'; 5 = 'Absolutely important') [Integer 1-5]. In case you are not aware of the item or not sure or don't want to answer, please select 'I don't know'.

1. Gut Bacteria and Diet: The last decade has brought us a greater understanding of the impact of our 'diet' on intestinal 'microbiota' (gut bacteria), and how changes in the 'microbiota' are associated with our health (cancer promotion and prevention).

2. Metabolism & exercise: Studies have shown that lifestyle behaviors may impact metabolism and cancer risk.

3. Chronic inflammation: Studies have shown that inflammation that becomes chronic or lasts for too long, often associates with the development and progression of cancer.

4. Substances causing cancer in the environment: Studies have shown that some environmental factors, called also carcinogens, increase the risk of developing cancer.

5. Prevention of cancer by the immune system and chemo treatments by using for instance vaccines, such as HPV vaccines, or preventive drugs for certain cancer types.

6 Cancer heredity & epigenetics: Studies have shown that cancers develop due to the accumulation of genetic (changes in the DNA sequence, some of which may be inherited) and epigenetic (changes not affecting the DNA sequence but its activity, that are non-inherited) alterations.

SCREENING & EARLY DIAGNOSIS

According to WHO, early detection of cancer greatly increases the chances for successful treatment. The 2 components of early detection of cancer are early diagnosis (or downstaging) and screening. Early diagnosis focuses on identifying patients with symptomatic disease as early as possible, while screening consists of testing a priori healthy individuals to identify those having incipient cancer lesions before any symptoms appear.

In this context, please rate the following topics on your perceived significance for further research in terms of their potential impact on cancer risk.

Scale from 1 to 5 (1 = 'Not important at all'; 2 = 'Slightly important'; 3 = 'Obviously important'; 4 = 'Strongly important'; 5 = 'Absolutely important') [Integer 1-5]. In case you are not aware of the item or not sure or don't want to answer, please select 'I don't know'.

1. Processes occurring before tumor development: The development of cancer is a multistep process in which normal cells gradually become malignant through progressive accumulation of molecular alterations.

2. Early cancer mechanisms: Cancer is a disease caused when cells divide uncontrollably and cooperate with other cells in their local environment which fosters tumor progression.

3. Blood tests for Early Detection: Specific blood tests are designed to identify tumor (bio)markers that may be found in the blood when some cancers are present before showing symptoms or being detected through conventional imaging approaches.

4. Technologies for Early Diagnosis: Numerous cancer-associated deaths occur from cancers for which we do not screen. To overcome this, new scalable and cost-effective technologies are developed to allow for the detection and diagnosis of cancers at an earlier stage when these are more responsive to treatments.

5. Personalized prevention and early screening: Everybody does not have the same risk of developing a cancer. Careful analysis of individual risk factors to adapt prevention and systematic screening to the risk level would increase the rate of early diagnosis

SENSITIVITY & RESISTANCE TO THERAPY

There are many types of cancer treatment that are administered according to the type of cancer that a patient has and how advanced it is. Some people with cancer will receive only one treatment, while a large proportion of patients are treated with a combination of therapies, such as surgery with chemotherapy and/or radiation therapy, immunotherapy, targeted therapy, or hormone therapy. However, efficacy is often limited by the emergence of resistance, and treatment may be accompanied by severe toxicities.

In this context, please rate the following topics on your perceived significance for further research in terms of their potential impact on cancer risk.

Scale from 1 to 5 (1 = 'Not important at all'; 2 = 'Slightly important'; 3 = 'Obviously important'; 4 = 'Strongly important'; 5 = 'Absolutely important') [Integer 1-5]. In case you are not aware of the item or not sure or don't want to answer, please select 'I don't know'.

1. Blood tests to show sensitivity and resistance to therapy: In the past two decades specific tests have been developed to customize the treatment plan for a cancer patient according to the sensitivity and resistance patterns that can be monitored by analyzing the patient's blood.
2. The biology of cancer cells (Immune system, stem cells, microenvironment, genetics, etc.): Studies have shown that not all cancer cells are created equal, and they have the capacity to remodel the cells around them. There are intrinsic differences in the proliferative and invasive capacity of cancer cells within the same patient, and immune cells in their environment also acquire specific properties.
3. New therapeutic approaches and drug delivery systems: The development of more specific anti-cancer drugs, new types of biological and immune-mediated therapies, combination of therapies with diverse mechanisms of action, and advanced drug delivery systems to target cancer cells more specifically, have the potential to improve cancer treatment for patients and reduce long-term effects.

CANCER & AGEING

Over time, the cells and tissues in our body accumulate damage, as a result of normal cell division and long-term exposures to chemicals, UV radiation, alcohol, or other sources of inflammation. Often this damage can be repaired by our bodies, but sometimes chronic damage can induce persistent inflammation that cooperates with

other factors to initiate cancer. As such, advancing age is the most important risk factor for cancer overall and many individual cancer types.
In this context, please rate the following topics on your perceived significance for further research in terms of their potential impact on cancer risk.
Scale from 1 to 5 (1 = 'Not important at all'; 2 = 'Slightly important'; 3 = 'Obviously important'; 4 = 'Strongly important'; 5 = 'Absolutely important') [Integer 1-5]. In case you are not aware of the item or not sure or don't want to answer, please select 'I don't know'.
1. Determinants of ageing and cancer: The incidence of most cancers increases with age as, for most adults, age is associated with chronic conditions, decreased efficacy of the immune system, cumulative exposure to risk factors (carcinogens), and tissue ageing with cell senescence, that is causally associated with cancer.
2. The cell biology of ageing and cancer: Aging is a complex phenomenon caused by the time-dependent loss of physiological organism functions including those that protect from cancer development.
3. Ageing and the process of ageing in cancer: Studies have shown that mechanisms of ageing are also found to occur in carcinogenesis. There is a need to better understand what aging and cancer development share and where the two processes diverge.
4. Influence of ageing on cancer interventions: Various studies support the hypothesis that cancer and/or cancer treatment is associated with accelerated biological aging. This is a key determinant of survivorship along with the long-term impact of cancer therapy on the biological aging of an individual.
5. Cancer complications and comorbidities: In older patients affected by cancer, it is key to consider not only the characteristics of the tumor but also pursue an integral geriatric assessment to systematically investigate factors that determine the well-being of patients. In this context, research suggests that we may be able to measure a biological age, which will be more precise than civil age to guide therapeutic choices when treating a cancer.

SURVIVORSHIP & QUALITY OF LIFE

Cancer and anticancer treatment are known to cause severe short- and long-term effects that compromise the quality of life of patients and cancer survivors. This creates the

need for surveillance for recurrence, monitoring of cancer- and treatment-related toxicity, and designing preventive and primary care interventions to attenuate the long-term and late physical and psychological effects of cancer and its treatment.
In this context, please rate the following topics on your perceived significance for further research in terms of their potential impact on cancer risk.
Scale from 1 to 5 (1 = 'Not important at all'; 2 = 'Slightly important'; 3 = 'Obviously important'; 4 = 'Strongly important'; 5 = 'Absolutely important') [Integer 1-5]. In case you are not aware of the item or not sure or don't want to answer, please select 'I don't know'.
1. Secondary cancers associated with treatment: Though it happens infrequently, patients may develop a secondary cancer as a result of the treatment received to treat the primary cancer.
2. Long-term Immune-related side effects: The effects of some cancer treatments can compromise some properties of the immune system, rendering patients vulnerable to viral and bacterial infections or causing autoimmune conditions.
3. Effects on reproductive functions and fertility: Cancer and its treatment can adversely impact reproductive function in both women and men. The effects of cancer treatment may lead to transient or permanent loss of fertility, sexual desire, and function.
4. Effects on the fitness of the heart and lungs and the hormone system: Both chemotherapy and radiation therapy to the chest can cause problems in the heart and lungs leading to potential cardiovascular or respiratory conditions that may be temporary or long-lasting.
5. Cancer treatments' effects on the nervous system: Chemotherapy and radiation therapy can cause long-term side effects on the brain, spinal cord, and nerves, and sometimes enhance pain sensitivity.
6. Comprehensive management and care in cancer survivors: For cancer survivors who are no longer in active treatment, their care needs include surveillance for recurrence, screening for the development of subsequent primary cancers, monitoring and intervention for the long-term and late physical and psychological effects of cancer and its treatment, management of comorbid medical conditions, as well as routine preventive and primary care.

DATA SHARING

All the significant breakthroughs in cancer understanding and therapy were driven by a methodological breakthrough. For example, progresses in microscopy revealed the

cellular nature of cancer, the discovery of X-rays and radium promoted radiation therapy, progress in chemical synthesis led to the use of chemotherapy, and developments in genetics and cell and molecular biology led to targeted and immune therapeutics. A new area has emerged with the ability to generate huge amounts of data through analysis of a given patient and to use artificial intelligence and machine learning (data sciences) to decipher the meaning of this information. Researchers need to get access to enormous amounts of data to generate new hypotheses and generate new therapeutic approaches, devices, and tools to improve the precision and results of cancer therapy.
In this context, please rate the following topics on your perceived significance for further research.
Scale from 1 to 5 (1 = 'Not important at all'; 2 = 'Slightly important'; 3 = 'Obviously important'; 4 = 'Strongly important'; 5 = 'Absolutely important') [Integer 1-5]. In case you are not aware of the item or not sure, please select 'I don't know'.

1. Generation of data: The development of data that may guide more precise therapeutic choices and generate more efficacy in treating cancer patients.
2. Use of data: Data whose analysis can inform on disease precise diagnosis, its heterogeneity, the existence of constitutive predisposing factors, and the ability of the patient to support and favorably respond to a given therapy.
3. Collection of data: With the tools of data sciences, researchers can collect and analyze data to identify common mechanisms in a large series of patients with similar diseases. With data sciences, the higher the number of patients analyzed, the most precise the analysis.
4. Quality of data: The efficacy of data sciences requires data standardization and interoperability to be re-used by multiple teams asking complementary questions.
5. Control of data sharing: Patient data sharing requires strict regulation to protect privacy (anonymization). While such a regulation is mandatory, it must also be organized in a manner that favors rather than prevents patient data sharing at the European level to support cancer research.
